# Accelerated fetal growth in early pregnancy and risk of preterm birth: a prospective cohort study

**DOI:** 10.1186/s12884-020-03458-x

**Published:** 2020-12-09

**Authors:** Evangelia Elenis, Anna-Karin Wikström, Marija Simic

**Affiliations:** 1 Department of Children’s and Women’s Health, Uppsala University, Uppsala University Hospital, SE-751 85 Uppsala, Sweden; 2 Clinical Epidemiology Unit, Department of Medicine Solna, Karolinska University Hospital and Institutet, Stockholm, SE-17176 Sweden

**Keywords:** Fetal growth, Preterm birth, Ultrasound, Early pregnancy, Induction of labor

## Abstract

**Background:**

Preterm birth (occurring before 37 completed weeks of gestation) affects 15 million infants annually, 7.5% of which die due to related complications. The detection and early diagnosis are therefore paramount in order to prevent the development of prematurity and its consequences. So far, focus has been laid on the association between reduced intrauterine fetal growth during late gestation and prematurity. The aim of the current study was to investigate the association between accelerated fetal growth in early pregnancy and the risk of preterm birth.

**Methods:**

This prospective cohort study included 69,617 singleton pregnancies without congenital malformations and with available biometric measurements during the first and second trimester. Estimation of fetal growth was based on measurements of biparietal diameter (BPD) at first and second trimester scan. We investigated the association between accelerated fetal growth and preterm birth prior to 37 weeks of gestation. The outcome was further stratified into very preterm birth (before 32 weeks of gestation) or moderate preterm birth (between 32 and 37 weeks of gestation) and medically induced or spontaneous preterm birth and was further explored.

**Results:**

The odds of prematurity were increased among fetuses with accelerated BPD growth (> 90th centile) estimated between first and second ultrasound scan, even after adjustment for possible confounders (aOR 1.36; 95% CI 1.20–1.54). The findings remained significant what regards moderate preterm births but not very preterm births. Regarding medically induced preterm birth, the odds were found to be elevated in the group of fetuses with accelerated growth in early pregnancy (aOR 1.34; 95% CI 1.11–1.63). On the contrary, fetuses with delayed fetal growth exhibited lower odds for both overall and spontaneous preterm birth.

**Conclusions:**

Fetuses with accelerated BPD growth in early pregnancy, detected by ultrasound examination during the second trimester, exhibited increased odds of being born preterm. The findings of the current study suggest that fetal growth in early pregnancy should be taken into account when assessing the risk for preterm birth.

**Supplementary Information:**

The online version contains supplementary material available at 10.1186/s12884-020-03458-x.

## Background

Preterm delivery, i.e., birth prior to 37 gestational weeks, complicates 4–13% of deliveries worldwide and is the predominant cause of neonatal morbidity and mortality, having an impact on approximately 15 million infants annually [1–3]. Preterm born infants have a higher risk for adverse outcomes primarily during the neonatal period; however, harmful effects have been described to extend even at later life stages, leading to attention deficit disorders and learning impairment in childhood [4, 5].

Previous research has described that the etiology and timing of preterm birth is multifactorial and several projects have focused on fetal growth, usually attempting to detect fetuses at increased risk of growth restriction. In contrast, only few reports have described the effect of increased fetal size at birth and spontaneous preterm onset of labor; in a population-based, as well as a hospital-based registry study performed in Sweden and Canada respectively, the authors demonstrated an increased risk for prematurity among fetuses with birthweight exceeding the expected [6, 7].

Despite the previous presumption that biological variation in fetal growth is minimal in early pregnancy and that abnormal fetal growth manifests at a later stage, there have been a number of studies showing the impact of fetal growth during early gestation on adverse pregnancy outcomes [8–11]. However, only few of them focused on accelerated fetal growth patterns among pregnancies with preterm birth. Lampl et al. followed fetal growth in 3927 pregnancies from gestational week 16 up to spontaneous birth and observed an increased risk for prematurity among fetuses with accelerated growth in the second trimester [11]. Pedersen et al. investigated fetal growth in 8215 early singleton pregnancies and reported an increased risk of preterm birth among fetuses with accelerated fetal growth, with results almost reaching statistical significance [10]. Hence, in order to overcome possible statistical challenges and enhance clinical relevance, we decided to perform a study with serial ultrasound measurements in a large, low risk obstetric population.

The primary aim of the present study was to investigate whether accelerated fetal growth, as assessed by BPD measurements, in the first half of pregnancy influenced the odds of preterm birth overall. The secondary aims were to further explore whether fetal growth differed between i) moderate and very preterm birth, as well as ii) spontaneous and medically induced preterm birth.

## Methods

### Study design and population

The study design is that of a longitudinal cohort study with prospectively collected data originating from a population-based obstetric database. The information gathered regard data on maternal, delivery and infant characteristics from all antenatal, ultrasound, delivery, and postnatal care units in the counties of Stockholm and Gotland in Sweden. Information about maternal reproductive history, lifestyle habits, height, weight, and state of health are usually recorded by midwives at the first antenatal visit and during pregnancy. Biometric measurements used in the study were collected from all ultrasound units in the region, and information about pre-gestational maternal diabetes and chronic hypertension, was obtained from standard delivery charts as well as diagnoses at discharge from the delivery hospital.

Singleton pregnancies (72,309) from the population area of the study with available data on biometric measurements in the first and early second trimester between January 1st, 2008 and October 22nd, 2014 were included in the study sample. Pregnancies with congenital malformations (*n* = 2495) [International Classification of Diseases tenth revision (ICD-10) codes Q00-Q99)] or stillbirths (*n* = 197) were excluded, resulting in a final study population of 69,617 singleton pregnancies.

### Exposure

The study exposure regarded differences in fetal biometric parameters, such as the biparietal diameter (BPD), measured by ultrasound scan during the first half of pregnancy. The Swedish Association for Obstetrics and Gynecology (SFOG) dictates that fetal BPD should be measured from the outer edge of the proximal parietal bone to the inner edge of the distal parietal bone at the level of thalami and septum pellucidum and performed by specially trained midwives according to a standardized protocol [8]. Firstly, we estimated gestational age based on biometrical measurements obtained at first trimester ultrasound scan (i.e. the combined ultrasound and biochemical screening test, CUB). Next, we related the observed fetal size at the second scan to the expected size estimated on the basis of the first trimester scan. At both first trimester (11 + 0 to 13 + 6 weeks of gestation) and second trimester (14 + 0 to 21 + 0 weeks of gestation) ultrasound examinations, gestational age was extrapolated from the formula by Selbing (gestational age = 58.65 + 1.07 x BPD + 0.0138 x ((BPD)^2^). Expected gestational age at second trimester scan was calculated by adding number of days between the two examinations to the observed gestational age at first trimester scan. The difference between observed and expected gestational age at second trimester scan, was expressed in z scores (z = $\frac{\chi -\mu }{\sigma }$, where μ equals the mean and σ equals the standard deviation). The individual z scores were transformed into centiles and fetuses with growth more than the 90th percentile were defined as having accelerated growth, while fetuses with growth between the 10th and 90th percentile were considered as appropriate and used as the reference group. Fetuses with growth smaller than expected (<10th percentile) were considered to be of delayed growth.

### Outcome

The outcome measure was overall prematurity (i.e. birth at < 37 + 0 weeks). Furthermore, we analyzed the odds for very preterm birth (birth at < 32 + 0 weeks) and moderate preterm birth (birth between 32 + 0 and 36 + 67 + 0 weeks). Preterm births with clinician-initiated obstetric interventions, as opposed to spontaneous preterm births, were defined as medically induced preterm births and included prelabor C-sections and induced labors.

### Covariates

Pregnancy characteristics collected from the woman’s medical records regarded maternal age at first antenatal visit (15–35 years or 36–55 years), maternal height (130–154 cm or 155–200 cm), BMI at early pregnancy (< or ≥ 30 kg/m^2^), primiparity (yes/no), smoking at early pregnancy (yes/no), use of in vitro fertilization (IVF) for the current pregnancy (yes/no), pre-gestational diabetes mellitus (yes/no), chronic hypertension (yes/no) and male fetal gender (yes/no). Pre-pregnancy diabetes mellitus was defined by the ICD-10 diagnosis codes O240, O241, while chronic hypertension was defined as treatment with antihypertensive medication at first antenatal visit and/or ICD-10 diagnosis code indicating chronic hypertension (O10, O11) developed at any time point before the 20th gestational week. ICD codes were provided by the responsible doctor at discharge from the hospital after delivery, while information regarding blood pressure measurements, proteinuria, and medication was provided by midwives at antenatal care or at the hospital before delivery. Information on fetal gender was collected from delivery charts. Maternal country of birth was divided into Sweden, other Nordic countries (i.e., Norway, Denmark, Finland, and Iceland), and non-Nordic countries. Information on paternal characteristics was not available in the database.

### Data analysis

The statistical software package SAS 9.4 (version 6.1; SAS, Cary, NC, USA) was used for the statistical analyses and a two-sided *p*-value below 0.05 was considered statistically significant. Pregnancy characteristics in categorical form were cross-tabulated with both exposure and outcome and compared with the use of chi-square test. In order to quantify the difference in growth in the three exposure groups the mean and dispersion index of the difference between observed and expected gestational age at second trimester scan for each of the three groups was calculated. The odds for accelerated growth (>90th percentile) or delayed growth (<10th percentile) were calculated based on discrepancy in fetal growth at early second trimester scan, using fetuses with appropriate growth (10th to 90th percentile) as the reference group. The odds of preterm birth were estimated by binary logistic regression analysis and expressed as crude and adjusted odds ratios (aORs) with 95% confidence intervals (CIs). Adjustments were made for maternal age (< or ≥ 35 years), maternal height (< or ≥ 155 cm), BMI (< or ≥ 30 kg/m^2^), non-Nordic origin (yes/no), primiparity (yes/no), smoking (yes/no), IVF (yes/no), pre-gestational diabetes mellitus (yes/no), chronic hypertension (yes/no) and male fetal gender. The selection of potential confounders recorded at the first antenatal visit, was based on directed acyclic graphs (DAGs) (Figure S1). Lastly, the multivariable risks and their distribution due to the presence of quantitative covariates (i.e. mean predictive value and standard deviation) were calculated for all categories of preterm birth based on a regression model for delayed, appropriate and accelerated fetal growth.

### Ethical approval

The study was approved by the Regional Ethical Review Authority in Stockholm, Sweden (Dnr 2014/2179–31/1). The need for written or oral informed consent for participation in the study was waived since all collected data were depersonalized prior to the analysis.

### Patient and public involvement

There was no patient or public involvement in the design, conduct, reporting or dissemination plans of our research study.

## Results

The characteristics of the study population according to gestational age at birth are presented in Table 1. Overall, 2838 (4%) of all pregnancies ended preterm; 2460 (3.5%) pregnancies ended between 32 and 37 weeks of gestation while 0.5% ended before 32 weeks of gestation. More specifically, 40.6% of overall preterm deliveries were initiated by clinicians (medically induced) and 59.4% started spontaneously. Mothers delivering preterm were more often pregnant through IVF, born outside the Nordic countries, were multiparous, overweight or obese, of short height or were treated for chronic hypertension.

**Table 1:** Baseline characteristics of mothers according to gestational age at birth. (*N* = 69,617 births)

Characteristics	Very preterm births^**a**^ (***n*** = 378)	Moderate preterm births^**b**^ (***n*** = 2460)	Term births^**c**^ (***n*** = 62,971)	Postterm births^**d**^ (***n*** = 3808)	***P***- value
**Maternal age older than 35 y**	197 (52.1)	1121 (45.6)	28,470 (45.2)	1789 (46.9)	0.009
**Short maternal height (< 155 cm)**	17 (4.5)	98 (3.9)	1508 (2.4)	57 (1.5)	< 0.0001
**Obesity (BMI ≥ 30 kg/m**^**2**^**)**	40 (10.9)	219 (9.4)	4055 (6.7)	271 (7.5)	< 0.0001
**Non- Nordic origin**	88 (25.8)	413 (19.6)	10,312 (18.4)	525 (15.9)	< 0.0001
**Primiparity**	182 (48.1)	1187 (48.2)	36,197 (57.5)	1629 (42.8)	< 0.0001
**Smoking**	15 (4.1)	82 (3.4)	1575 (2.5)	82 (2.2)	0.007
**In vitro fertilization (IVF)**	45 (11.9)	228 (9.3)	3993 (6.3)	168 (4.4)	< 0.0001
**Chronic hypertension**	11 (2.9)	51 (2.1)	556 (0.9)	27 (0.7)	< 0.0001
**Pre-gestational diabetes mellitus**	1 (0.2)	28 (1.1)	116 (0.2)	0	< 0.0001
**Male fetal gender**	208 (55.0)	1334 (54.2)	31,959 (50.7)	2028 (53.3)	< 0.0001

^a^Very preterm birth: gestational age less than 32 weeks

^b^Moderate preterm birth: gestational age between 32 + 0 and 36 + 6

^c^Term birth: gestational age between 37 + 0 and 41 + 6

^d^Postterm birth: gestational age 42 weeks or more

Based on the information on biometric measurements, 7091 (10.1%) of fetuses had BPD growth more than the 90th percentile at the second trimester ultrasonic scans. In 55,571 (79.8%) of fetuses, the growth was between 10th and 90th percentile, while 10.1% of fetuses had a BPD growth smaller than expected (< 10th percentile). Mothers whose fetus had exhibited accelerated fetal growth at second trimester were more often younger and multiparous compared to those whose fetus had appropriate fetal growth. Male fetuses at second trimester were more often larger in size than female fetuses (Table 2).

**Table 2:** Baseline characteristics according to fetal growth in early pregnancy. (*N* = 69,617 births)

Characteristics	Accelerated fetal growth^**a**^ (***n*** = 7091)	Appropriate fetal growth^**b**^ (***n*** = 55,571)	Delayed fetal growth^**c**^ (***n*** = 6955)	***P***- value
**Maternal age older than 35 y**	3117 (43.9)	25,108 (45.2)	3352 (48.2)	< 0.0001
**Short maternal height (< 155 cm)**	143 (2.0)	1367 (2.4)	170 (2.4)	0.13
**Obesity (BMI ≥30 kg/m**^**2**^**)**	440 (6.5)	3658 (6.9)	487 (7.3)	0.28
**Non- Nordic origin**	1092 (17.7)	9043 (18.4)	1203 (18.9)	0.64
**Primiparity**	3409 (48.1)	31,269 (56.3)	4517 (64.9)	< 0.0001
**Smoking**	177 (2.5)	1538 (2.5)	210 (3.0)	0.03
**In vitro fertilization (IVF)**	443 (6.2)	3649 (6.5)	342 (4.9)	< 0.0001
**Chronic hypertension**	62 (0.9)	523 (0.9)	60 (0.9)	0.45
**Pre-gestational diabetes mellitus**	12 (0.2)	116 (0.2)	17 (0.2)	0.75
**Male fetal gender**	4988 (70.4)	28,179 (50.7)	2362 (33.9)	< 0.0001
**Difference in growth** **mean (dispersion index)**	5.14 (0.45)	−0.63(−7.29)	−6.56 (−0.34)	< 0.0001

^a^Accelerated fetal growth at second trimester scan was defined as BPD measurement at the 90th percentile, or more of the expected for gestational age

^b^Appropriate fetal growth at second trimester scan was defined as BPD measurement between 10th – 90th percentile of the expected for gestational age

^c^Delayed fetal growth at second trimester scan was defined as BPD measurement at the 10th percentile, or less of the expected for gestational age

^d^Difference between observed and expected gestational age at second trimester scan

Compared to fetuses with appropriate growth at early second trimester ultrasound, fetuses with accelerated growth had a 40% increased risk for prematurity, both what regards moderate as well as very preterm birth. The association between fetal growth and overall and moderate preterm birth remained unchanged despite adjusting for maternal characteristics, pre-pregnancy diabetes, chronic hypertension and fetal gender (Table 3). However, the risk for very preterm birth did not reach statistical significance after adjustment.

**Table 3:** Growth in early pregnancy and risk of preterm birth, very preterm and moderate preterm birth. (*N* = 2838 births)

Growth at second trim scan	All preterm births (***n*** = 2838)			Very preterm births (***n*** = 378)			Moderate preterm births (***n*** = 2460)		
	No. of women	Odds ratio (95% CI)		No. of women	Odds ratio (95% CI)		No. of women	Odds ratio (95% CI)	
		Crude	Adjusted^**d**^		Crude	Adjusted^**d**^		Crude	Adjusted^**d**^
**Accelerated fetal growth**^**a**^	389	1.39 (1.25–1.56)^*^	1.36 (1.20–1.54)^*^	52	1.38 (1.01–1.86)^**^	1.33 (0.96–1.83)^**^	337	1.39 (1.24–1.57)^*^	1.36 (1.18–1.55)^*^
**Appropriate fetal growth**^**b**^	2215	Ref	Ref	295	Ref	Ref	1920	Ref	Ref
**Delayed fetal growth**^**c**^	234	0.85 (0.73–0.96)^*^	0.84 (0.71–0.97)^*^	31	0.84 (0.58–1.21)^***^	0.73 (0.47–1.12)^***^	203	0.84 (0.72–0.97)^*^	0.85 (0.73–1.00)^*^

^a^Accelerated fetal growth at second trimester scan was defined as BPD measurement at the 90th percentile, or more of the expected for gestational age

^b^Appropriate fetal growth at second trimester scan was defined as BPD measurement between 10th -90th percentile of the expected for gestational age

^c^Delayed fetal growth at second trimester scan was defined as BPD measurement at the 10th percentile, or less of the expected for gestational age

^d^Adjusted OR for maternal age older than 35 yrs., short maternal height < 155 cm, BMI ≥ 30 kg/m^2^, non-Nordic origin, primiparity, smoking, IVF, pre-gestational diabetes mellitus, chronic hypertension and male fetal gender

^*^p < 0.0001

^**^p < 0.05

^***^p > 0.05

Medically induced labor was observed in 40.6% of preterm births with the odds of prematurity being increased among fetuses with accelerated growth compared to fetuses with appropriate growth at second trimester scan. Even after adjustment for relevant confounders, the odds remained elevated (Table 4).

**Table 4:** Growth in early pregnancy and risk of preterm birth either spontaneous or medically induced. (*N* = 2838 births)

Growth at second trim scan	Spontaneous preterm birth (***n*** = 1687)			Medically induced preterm birth (***n*** = 1151)		
	No. of women	Odds ratio (95% CI)		No. of women	Odds ratio (95% CI)	
		Crude	Adjusted^**d**^		Crude	Adjusted^**d**^
**Accelerated fetal growth**^**a**^	234	1.39 (1.21–1.61)^*^	1.35 (1.15–1.58)^*^	155	1.37 (1.15–1.63)^*^	1.34 (1.11–1.63)^**^
**Appropriate fetal growth**^**b**^	1325	Ref	Ref	890	Ref	Ref
**Delayed fetal growth**^**c**^	128	0.78 (0.64–0.92)^*^	0.78 (0.63–0.96)^*^	106	0.95 (0.77–1.16)^***^	0.91 (0.72–1.15)^***^

^a^Accelerated fetal growth at second trimester scan was defined as BPD measurement at the 90th percentile, or more of the expected for gestational age

^b^Appropriate fetal growth at second trimester scan was defined as BPD measurement between 10th - 90th percentile of the expected for gestational age

^c^Delayed fetal growth at second trimester scan was defined as BPD measurement at the 10th percentile, or less of the expected for gestational age

^d^Adjusted OR for maternal age older than 35 yrs., short maternal height < 155 cm, BMI ≥ 30 kg/m^2^, non-Nordic origin, primiparity, smoking, IVF, chronic hypertension, pre-gestational diabetes mellitus and male fetal gender

^*^p < 0.0001

^**^p < 0.01

^***^p = 0.05

On the other hand, 6955 fetuses were smaller than expected at second trimester scan (growth less than 10th percentile). Among them, 234 fetuses were born preterm (31 were born very preterm and 203 moderate preterm). Fetuses that experienced delayed growth in early pregnancy, had a decreased risk for preterm birth (aOR 0.84; 95% CI 0.71–0.97), an effect seen mostly among spontaneously preterm born infants (Tables 3 and 4).

Lastly, after adjusting for relevant confounders the mean predictive value of preterm birth for fetuses with delayed, appropriate and accelerated growth was estimated to be 0.031, 0.038 and 0.053 respectively (Supplementary Table).

## Discussion

In this registry-based study, we have demonstrated that early accelerated fetal growth, reflected by an increase in BPD measurement estimated by ultrasound, was associated with increased odds of preterm birth. The effect estimates remained unchanged even after stratification according to timing, as well as to type of preterm birth (i.e. very preterm vs moderate preterm birth and medically induced vs spontaneous preterm birth). These findings underline the importance of accelerated fetal growth in early pregnancy and the potential association to complications later in pregnancy.

In accordance with our study, Pedersen et al. demonstrated an increased risk for prematurity among fetuses with accelerated growth; OR 3.27 (95% CI, 0.99–10.73) for very preterm births 22–33 weeks and OR 2.30 (95% CI, 1.15–4.59) for moderate preterm births [10]. Similarly to our study, their population included a relatively small number of pregnant women suffering from very preterm birth, which could partly explain why the results did not reach statistical significance (*p* = 0.074) [10]. Additionally, the association between increased birthweight and elevated risk of preterm birth has been previously described, but without focus on growth in early pregnancy [6, 7]. In the cohort of 1 million births recorded in the Swedish Medical Birth Register, Morken et al. observed increased risk for birth between 34 and 36 weeks of gestation (OR 1.6, 95% CI 1.5–1.7) among infants with larger birthweight than the population mean [7]. Similarly, Gaillard et al., observed an increased risk for overall and spontaneous preterm birth among fetuses with larger head circumference in second trimester [12]. On the contrary, in the study performed by Partap et al., the authors observed an inverse relationship between growth velocity of femur length in second trimester and risk of spontaneous preterm birth [13]. Another finding of our study was the decreased risk for prematurity observed among fetuses with delayed fetal growth at the ultrasound scan of the second trimester. Despite adjusting for chronic hypertension, preeclampsia and other potential risk factors for medically induced prematurity, the risk of prematurity for fetuses with delayed growth remained nevertheless lower compared to the reference group (i.e., fetuses with normal growth). One possible explanation of this finding is the exclusion of pregnancies with fetal aneuploidy, congenital anomalies and fetal demise from the study population according to study design. Since the prior conditions have been associated to delayed fetal growth [14–16], it is not surprising that this exclusion might deflate the expected rates of preterm birth in this subgroup of fetuses.

The incidence of preterm birth estimated in our study is in accordance with the reported national rates in Sweden and other Nordic countries [7, 17, 18]. However, in our study, 40.6% of preterm births were classified as medically initiated births, compared to 35 and 25% reported in previous Swedish studies [7, 17]. These variations could be the result of methodological differences in study design such as different cut-offs of preterm birth employed, or different outcome definitions such as that of premature preterm rupture of membranes (pPROM) [7, 17]. Furthermore, the higher rates of medically induced preterm births observed in our study could reflect increasing rates of medical intervention over time, a trend that has also been reported in other settings [18–21].

Although the exact pathophysiological mechanism behind our findings still remains unknown, a number of possible hypotheses associated to the onset of labor have been proposed. One of the theories to explain the timing of birth refers to prelabor mechanical distention and stretching of the myometrium [22]. The overdistention of the uterus leads to increased expression of contraction-associated proteins (such as connexin-43), and initiate the necessary biochemical steps towards coordinated and forceful contractions [23–25]. Based on the physiology of uterine contractions, it is thus reasonable to assume that accelerated fetal growth could increase the distension demands posed on the uterus to a greater degree than during normal fetal growth and subsequently trigger spontaneous preterm labor. The theory is also in line with the contrasting finding of preterm birth and delayed fetal growth. One can only assume that fetuses smaller than expected, do not grow at the same velocity and probably do not reach equally large birthweights as fetuses with accelerated growth, decreasing thereby the risk of triggering or activating the labor system at a preterm stage.

One of the major strengths of our study is the accurate estimation of gestational age based on ultrasonographic measurements at the first trimester of pregnancy and not on the self-reported last menstrual period of the participating women which can often be unreliable. Furthermore, we attempted to explore growth velocity based on the measurements of only one biometric characteristic (i.e., BPD), since it was the only biophysical parameter that was readily available both at first and second trimester scan. The latter, in addition to the possibility of calculating gestational age solely based on BPD measurements also justifies why the formula by Selbing was employed, enabling us at the same time to avoid uncertainty introduced by utilizing different calculation methods. Another major strength lies on the large sample size with detailed information collected prospectively, which limits the risk for recall bias. The large cohort size in the study, made it possible to stratify the analysis in spontaneous and medically induced groups, as well as in moderately preterm and very preterm groups and therefore explore rare outcomes. Furthermore, Sweden has a long tradition of valid registries entailing routinely collected, comprehensive and accurate data especially in the perinatal field. In addition, the publicly funded healthcare system of the country enables equal access to prenatal healthcare and early identification and possibly preventive intervention against preterm birth. That makes Sweden an appropriate model country to explore the research question. Lastly, since all ultrasound units in Sweden follow the same professional standards, we can only assume that the performance variation of the ultrasound examiners is restricted [8].

The present study is however not void of limitations. First and foremost, we lack information on the paternal characteristics, which along with the maternal might influence fetal growth. We also lack information on potential risk factors associated to the outcome (i.e. preterm birth) such as prior preterm birth, cervical length, uterine fibroid or malformations and genitourinary infections. However, all of these covariates are not anticipated, according to the literature, to be unevenly distributed between the groups of interest, i.e. the reference group of fetuses with appropriate growth and the comparison group of fetuses with accelerated growth. Furthermore, as we were obliged to restrict our population to pregnancies where CUB was performed and a fraction of the population did not perform first trimester sonographic scans, a potential selection bias related to advanced maternal age cannot entirely be ruled out. We have therefore tried to account for it by adjusting for advanced maternal age in the logistic regression analysis. However, despite the fact that the CUB scan is optional in Sweden, many women choose nevertheless to undergo ultrasound examination both in the first and second trimester making our results applicable in the clinical setting. There is of course the risk that fetal growth velocity was already affected at the time of the dating of the pregnancy (gestational week 11–14), on which the calculation of gestational age was based. By including in the performed risk analyses detailed information on maternal, pregnancy and fetal characteristics, known to affect both first-trimester growth and the risk of preterm birth [26–31] that scenario was accounted for. Finally, our population comes mainly from the urban area of the capital of Sweden (Stockholm) and its composition corresponds to that of other large cities, potentially affecting the generalizability of our findings in the wider birthing population.

## Conclusion

Accelerated fetal growth during early gestation is associated with an increased risk of preterm birth overall, as well as with medically induced and spontaneous preterm birth. There is currently no recommendation on the management of pregnancies with accelerated fetal growth early in pregnancy. Identification of pregnancies at risk could allow proper interventions such as counselling on maternal nutrition and physical activity or even introduction of more appropriate surveillance considering the risk for prematurity. Further research on the topic is therefore warranted.

## Supplementary Information

**Additional file 1: Figure S1.** Directed acyclic graph (DAG) showing the relation between covariates included in the analyses and exposure (accelerated early fetal growth) and outcome (preterm birth).

**Additional file 2: Supplementary Table.** Growth in early pregnancy and multivariable risk of overall preterm birth and its subcategories (very preterm, moderate preterm, spontaneous preterm and medically induced preterm birth).

## Data Availability

The datasets generated and analysed during the current study are not publicly available and cannot be uploaded at any website due to the risk of compromising the individual privacy of participants. On the other hand, the data is available from the responsible department upon reasonable request. Any interested parties are welcome to contact the authors, who will then fill out the agreements necessary when sharing data and after approval by the Regional Ethics Board in Stockholm, Sweden. All data regarding the current study are available on request to the Department of Women’s and Children’s Health, Karolinska Institutet.

## Abbreviations

ICD: International Statistical Classification of diseases–10th edition
BPD: Biparietal diameter
OR: Odds ratio
CI: Confidence interval
BMI: Body mass index
IVF: In vitro fertilization
CUB: Combined ultrasound and biochemical screening test
