# Supplementary figures and images for: Accelerated fetal growth in early pregnancy and risk of preterm birth: a prospective cohort study
Source: BMC Pregnancy Childbirth. 2020 Dec 9;20:764. doi: 10.1186/s12884-020-03458-x (PMC7724842; doi:10.1186/s12884-020-03458-x)

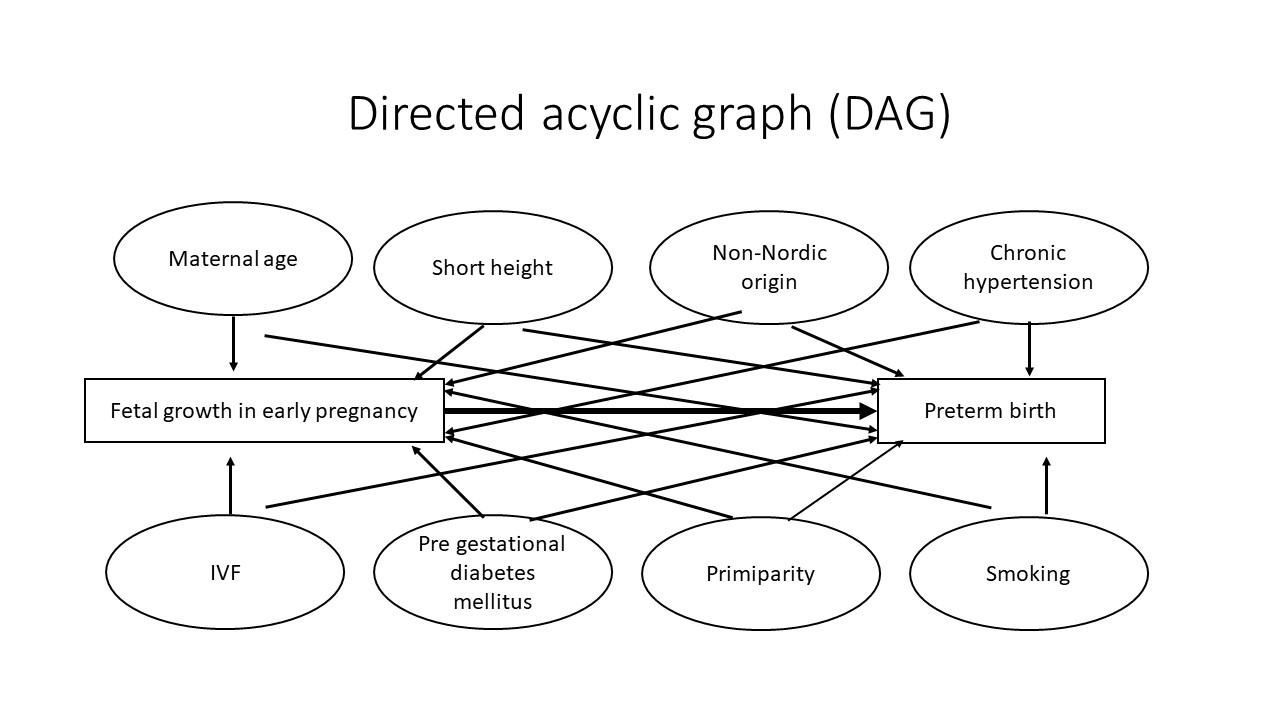

Supplement: Supplementary file 1 — Additional file 1: Figure S1. Directed acyclic graph (DAG) showing the relation between covariates included in the analyses and exposure (accelerated early fetal growth) and outcome (preterm birth). [file 12884_2020_3458_MOESM1_ESM.jpg]
