# Supplementary material for: Accelerated fetal growth in early pregnancy and risk of preterm birth: a prospective cohort study
Source: BMC Pregnancy Childbirth. 2020 Dec 9;20:764. doi: 10.1186/s12884-020-03458-x (PMC7724842; doi:10.1186/s12884-020-03458-x)
Supplement: Supplementary file 2 — Additional file 2: Supplementary Table. Growth in early pregnancy and multivariable risk of overall preterm birth and its subcategories (very preterm, moderate preterm, spontaneous preterm and medically induced preterm birth). [file 12884_2020_3458_MOESM2_ESM.docx]

**Supplementary Table.** Growth in early pregnancy and multivariable risk of overall preterm birth and its subcategories (very preterm, moderate preterm, spontaneous preterm and medically induced preterm birth).

|  | Overall preterm birth | Moderate preterm birth | Very preterm birth | Spontaneous preterm birth | Medically induced preterm birth |
| --- | --- | --- | --- | --- | --- |
| Mean predictive value(Standard deviation) | | | | | |
| Accelerated fetal growth | 0.053(0.02) | 0.048(0.21) | 0.007(0.09) | 0.033(0.18) | 0.022(0.15) |
|  |  |  |  |  |  |
| Appropriate fetal growth | 0.038(0.01) | 0.035(0.18) | 0.005(0.07) | 0.024(0.16) | 0.016(0.13) |
|  |  |  |  |  |  |
| Delayed fetal growth | 0.031(0.01) | 0.029(0.17) | 0.004(0.07) | 0.018(0.13) | 0.015(0.12) |
